# Formation of Predictive-Based Models for Monitoring the Microbiological Quality of Beef Meat Processed for Fast-Food Restaurants

**DOI:** 10.3390/ijerph192416727

**Published:** 2022-12-13

**Authors:** Olja Šovljanski, Lato Pezo, Ana Tomić, Aleksandra Ranitović, Dragoljub Cvetković, Siniša Markov

**Affiliations:** 1 Faculty of Technology Novi Sad, University of Novi Sad, Bulevar cara Lazara 1, 21000 Novi Sad, Serbia; 2 Institute for General and Physical Chemistry, Studentski trg 12/V, 11000 Belgrade, Serbia

**Keywords:** beef minced meat, microbiological analysis, ISO standards, prediction models, meat microbiology

## Abstract

Consumption of raw or undercooked meat is responsible for 2.3 million foodborne illnesses yearly in Europe alone. The greater part of this illness is associated with beef meat, which is used in many traditional dishes across the world. Beneath the low microbiological quality of beef lies (pathogenic) bacterial contamination during primary production as well as inadequate hygiene operations along the farm-to-fork chain. Therefore, this study seeks to understand the microbiological quality pathway of minced beef processed for fast-food restaurants over three years using an artificial neural network (ANN) system. This simultaneous approach provided adequate precision for the prediction of a microbiological profile of minced beef meat as one of the easily spoiled products with a short shelf life. For the first time, an ANN model was developed to predict the microbiological profile of beef minced meat in fast-food restaurants according to meat and storage temperatures, butcher identification, and working shift. Predictive challenges were identified and included standardized microbiological analyses that are recommended for freshly processed meat. The obtained predictive models (an overall *r*^2^ of 0.867 during the training cycle) can serve as a source of data and help for the scientific community and food safety authorities to identify specific monitoring and research needs.

## 1. Introduction

The presence of microbes in food is a major issue in the food industry. This problem arises as a global issue because it poses a constant threat to health. For example, foodborne illnesses related to food-producing animals affect millions of people every year and cause approximately 5000 deaths [1]. In order to solve this problem, it is necessary to constantly develop procedures that would minimize the occurrence and survival of microbiological contamination in food and food preparation systems. This problem is reflected in the fact that a large number of microorganisms can survive the effects of chemicals allowed for use in food, as well as physicochemical processes currently used in the industry [2]. Food safety depends on appropriate state regulations, but also requires the proper application of legal regulations, such as constant training of people involved in food manipulation. Basic rules of food hygiene are essential, but they are still missing in many steps of food processing. 

Meat is an easily spoiled product with a short shelf life [3]. Some very popular dishes across the world are meat-based, while a minced version of meat is essential for making traditional foods in some parts of Europe [2]. Ground or minced meat, like all other raw meat products, is a water-based substrate, with approximately 99% water. Additionally, it consists of an acceptable pH value and nutritional composition which can cause microbial proliferation and development contamination [4]. After initial contamination, microbial growth is rapid, and the number of viable cells is exponentially increased in a short time [5]. Many factors can affect meat quality, such as storage temperature, moisture, oxygen availability, packaging, microbiological contamination, etc. [6]. Several research groups have reported that temperature represents the most influential factor for meat quality and safety. More precisely, inadequate storage, manufacturing, and transport temperature, as well as variability in meat temperature profile, will increase the shelf life of meat [7]. It can be summarized that storage temperature, and consequently meat temperature, is directly connected to the risk of foodborne diseases [8]. Besides the fact that muscle from healthy animals is sterile, poor hygiene practices during slaughtering and cutting can change the microbiological profile of processed meat [9]. Microbial contamination exists primarily on the meat’s surface, which is exposed to external conditions but also can be spread to the entire production process. 

The most potent phases for microbial spreading in meat processing are the mincing and mixing phases [10,11]. The dispersion of microbiological contamination during these phases is of major concern for quantitative microbiological risk assessment. For example, eating raw or undercooked minced meat in fast-food restaurants has been reported as the cause of large outbreaks of salmonellosis and *Escherichia coli* infection [12,13,14,15]. Pathogens such as *Salmonella* spp. and *E. coli* may be present in the gastrointestinal tract of cattle [16], which can endanger the microbiological quality of meat after the slaughtering process [17]. As one of the faecal indicators, *E. coli* represents an illness-causing agent, and much effort has been invested in risk assessment of the public health impact of this bacterium, especially of *E. coli* O157:H7 in ground beef [18]. Chilled raw beef is a major source of pathogenic *E. coli*, and it has been supposed that such organisms in the faeces of cattle are spread to meat during processing steps [19]. Due to this fact, this bacterium is recognized worldwide as one of the main hygiene criteria in the meat industry [3]. Along with *E. coli*, *Staphylococcus aureus* is also one of the impacts of meat processing. Namely, Mohamed [2] reported that the presence of *S. aureus* can be expected as meat contamination, because this bacterium represents normal flora in humans and its presence in foods indicates inadequate butcher handling. As one of the bacteria that can grow and produce toxins under different environmental and nutritional conditions, *S. aureus* can cause food poisoning by enterotoxins, while meat and meat products represent frequently incriminated foods in staphylococcal food poisoning [20]. As two main food safety criteria, *Salmonella* spp. and *Listeria monocytogenes* are recognized for many types of food products and can be transferred to contaminated raw or undercooked red meats [21,22]. In the last few years, there has been an increasing trend in reporting outbreaks and sporadic cases associated with meat and meat products contaminated with these bacteria [22]. Aside from these specific bacteria, which are monitored in meat and meat products, one of the mainly followed parameters of a hygienic routine in meat processing is mesophilic and aerobic bacteria. In many reports, a high level of contamination of meat with this group of bacteria is an unsatisfactory parameter indicating the need for better hygiene practices during meat processing, as well as choice and/or raw material improvements [10,11,23,24]. Furthermore, lactic acid bacteria (LAB) are consequently present as contamination microflora of meat after the slaughtering process. These bacteria may reach a significant number in packaged and refrigerated minced meat. Depending on the LAB genera, this bacterial group can be involved in meat product preservation and development of desirable sensory characteristics (homofermentative LAB) or cause meat spoilage (in general heterofermentative LAB) [25]. Other, but less monitored, specific spoilage bacteria for red meat and meat products are *Pseudomonas* and *Clostridium* representatives, as well as Enterobacteriaceae, *Shewanella putrefaciens*, and *Brochothrix thermosphacta* [26].

Monitoring of the microbiological profile is very difficult during each step in the supply chain, although there is adequate standardized microbiological analysis. Obtaining results requires a lot of time, while the meat is usually transported immediately after processing to the retail phase. Due to this fact, many meat industry producers are open to the implementation of intelligent systems that can predict the microbiological quality of the meat and meat products based on the experimental data collected over time. Using predictive microbiologically techniques, meat producers can decrease the cost and time spent on the microbiological analysis and also have a better picture of microbial contamination, growth, mortality, and survival of microorganisms [27]. In recent years, many prediction models have been utilized for a predictive microbiological profile in different scientific and industrial fields, but using artificial neural network (ANN) modelling is a widely accepted tool for providing an empirical explanation of microbial behaviour. Moreover, ANN represents an essential base for handling complex responses with nonlinearities and interactions between decision variables [28].

Therefore, the formation of a system for long-time monitoring of the microbiological quality of beef meat based on the predictive capabilities of the ANN models was tested, using standardized microbiological analysis, as well as meat temperature, storage temperature, butcher working shift, and sampling weekday (equally with weekday for primary production and distribution). Sampling was conducted for three consecutive years (2019–2021) for the same meat production after the mincing and mixing process. The obtained experimental data were served for mathematical modelling and determination of the relative influence of independent variables on the microbiological profile of beef minced meat.

## 2. Materials and Methods

### 2.1. Sampling

The minced meat samples were obtained from a local butcher specializing in beef, located in Novi Pazar, Republic of Serbia. All processed meat batches were transported at 4 °C to the fast-food restaurant in Novi Sad, Republic of Serbia. Samples represent ground meat without bones that is minced and contains less than 1% salt. Approximately one hundred grams of minced meat was obtained from each batch immediately after transport and placed in a sterilized sampling box. The sample collection was conducted for three consecutive years (2019–2021, January–June, every Monday, Wednesday, and Friday) for each batch transported to the restaurant after the phase of mincing and mixing.

### 2.2. Microbiological Analysis

The microbiological profile of minced meat samples was determined following standard methods presented in Table 1. Although the Rulebook on general and specific food hygiene requirements in any phase of food production, processing, and trade in the Republic of Serbia (Official Gazette RS, No. 72/2010) recommend only two analyses—determination of aerobic and mesophilic bacteria and *Escherichia coli* [29]—microbiological analysis in this study was expanded with the examination indicated in the *Guide to Microbiological Criteria for Food* [30] and previous experiences of the butcher. The obtained results were compared to the allowable values for every type of food. Briefly, all results are presented as the number of a colony in a gram of samples (log CFU/g), except for the determination of *Salmonella* spp. or *L. monocytogenes*, where the absence of the bacterium is required. All analyses were performed in five repetitions.

According to the Rulebook, the obtained results can be classified as satisfactory (result is below m-value), acceptable (result is between m- and M-value), or unsatisfactory (result is above M-value). The m- and M-value represent defined limit values, minimum and maximum for each analysis separately, and depend on the type of food. Briefly, to determine compliance with food safety or hygiene criteria for one batch of food, it is mandatory to take an adequate number of samples in a few repetitions (e.g., five). If the results of the microbiological testing are less than or equal to the limit (<m-value), the tested sample can be evaluated as satisfactory. In the case when the maximum allowed results are between m- and M-value and all other results are less than or equal to the m-value, the sample can be defined as acceptable. On the other hand, when one or more results exceed the M-value, a sample is unsatisfactory. If one or more tested microorganisms are deemed acceptable and if the results for other tested microorganisms are consistent, then the sample is considered acceptable. If one or more results for a particular parameter are assessed as non-compliant, then the sample is unsatisfactory [30].

### 2.3. Statistical Analysis

As one of the potential solutions for long-time monitoring of microbiological criteria in food production processes, advanced mathematical tools can be included in the interpretation of the obtained results. In view of practical application, this type of mathematical tool can potentially decrease some crucial time-consuming and economic parameters of microbiological profiling of food samples, such as the number of samples, frequency of analyses, etc. Therefore, this study used a mathematical modelling approach to evaluate the obtained results of microbiological testing. Descriptive statistics were used for the collected data, while mathematical modelling was performed using STATISTICA 10.0 software (StatSoft Inc., Tulsa, OK, USA, 2010). The independent variables used for ANN modelling were: meat temperature, storage temperature, butcher identification (butcher A, B, C, and D), and sampling weekday (Monday, Wednesday, and Friday)—equally with weekday for primary production and distribution, while the output variables were the microbiological profile of beef minced meat. Butchers (meatmen) are permanent employees in the local butcher located in Novi Pazar, Republic of Serbia. In order to classify them, each butcher is marked with A, B, C, or D instead of their names, which is in accordance with their shifts. The selected four independent variables were chosen due to their reproducibility and traceability in any other butcher without special equipment or investments. In this way, processing and sampling parameters as well as a human influence were included in this study. In the whole process, butchers A, B, C, and D were the same persons working in continuous shifts at the butcher. The storage temperature was determined by an inside/outside thermometer (Carl Roth, Karlsruhe, Germany), while meat temperature was monitored by a penetration laboratory thermometer (Carl Roth, Karlsruhe, Germany). Principal component analysis (PCA) was selected as a tool to discover the possible correlations among measured parameters (MAB, *E. coli*, *Salmonella* spp., *S. aureus*, and LAB) and to classify objects into groups in the factor space.

#### 2.3.1. Artificial Neural Network (ANN) Modelling

The ANN model was built as a three-layer perceptron model, having input, hidden, and output layers. The data were normalized prior to the ANN modelling, in order to facilitate the improvement of the ANN model’s conduct [37]. The experimentally derived data were randomly parted into training, cross-validation, and testing datasets, prior to ANN model calculation. The training dataset was used for the learning cycle and the investigation of the optimal number of neurons in the hidden layer and the calculation of the weight coefficient associated with each network neuron. The Broyden–Fletcher–Goldfarb–Shanno (BFGS) procedure was employed for the solving of the unconstrained nonlinear optimization problem during the ANN model’s development. The effective ANN training was accomplished when learning and cross-validation curves reached zero [38]. Coefficients assigned to the hidden and the output layers (weights and biases) were presented in matrices *W*_1_ and *B*_1_ as well as *W*_2_ and *B*_2_, respectively. Equation (1) presents the ANN by using matrix notation.

(1) $$Y=f_{1}(W_{2}\cdot f_{2}(W_{1}\cdot X+B_{1})+B_{2})$$

where *Y* is the matrix of the output variables, *f*_1_ and *f*_2_ are transfer functions in the hidden and output layers, respectively, and *X* is the matrix of input variables. The *W*_1_ and *W*_2_ are determined during the ANN learning cycle using minimization of the error between the network and experimental outputs, according to the sum of squares (SOS) and the BFGS algorithm used to speed up and stabilize convergence. The coefficients of determination were used to check the performance of the gained ANN model [39,40]. 

A numerical verification of the obtained models in the previous step was tested using the coefficient of determination (*r*^2^), reduced chi-squared (χ^2^), mean bias error (MBE), root mean square error (RMSE), and mean percentage error (MPE) using Equations (2)–(5) [37,41]. A statistical test that determines how well sample data fit a distribution from a population with a normal distribution, “the goodness of fit” was evaluated for the obtained mathematical models based on the mentioned numerical verification.

(2) $$\chi^{2}=\frac{\sum_{i=1}^{N}{\left(x_{\exp,i}-x_{pre,i}\right)}^{2}}{N-n}$$

(3) $$RMSE={\left[\frac{1}{N}\cdot \sum_{i=1}^{N}{\left(x_{pre,i}-x_{\exp,i}\right)}^{2}\right]}^{1/2}$$

(4) $$MBE=\frac{1}{N}\cdot \sum_{i=1}^{N}\left(x_{pre,i}-x_{\exp,i}\right)$$

(5) $$MPE=\frac{100}{N}\cdot \sum_{i=1}^{N}\left(\frac{\left|x_{pre,i}-x_{\exp,i}\right|}{x_{\exp,i}}\right)$$

In Equations (2)–(5), *x_exp,i_* represents the experimental values, and *x_pre,i_* represents the predicted values obtained by calculating the model for these measurements. *N* and n are the number of observations and constants, respectively.

#### 2.3.2. Global Sensitivity Analysis

Yoon’s interpretation method was used to determine the relative influence of independent variables on the microbiological profile of beef minced meat [42]. This method was applied based on the weight coefficients of the developed ANN.

## 3. Results

Minced meat represents a good medium for microbiological growth, which can primarily be spotted on the meat’s surface but is also distributed into the meat product during the mincing and mixing process [40]. Therefore, the first steps included the determination of the microbiological profile of meat samples using standardized methods. The summarized microbiological quality of meat samples is shown in Table 2 based on the distribution of the obtained results on the satisfactory, acceptable, and unsatisfactory levels presented in percentages. The obtained results are connected with allowable limit values according to the *Guide to Microbiological Criteria for Minced Meat Samples* [30]. All analyses presented in Table 2 are performed using protocols from proper ISO standards (see Table 1).

**Figure 1:**
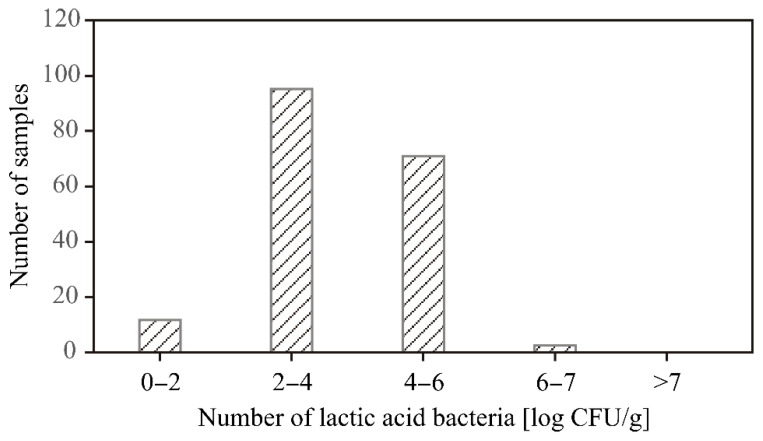
Frequency plot of lactic acid bacteria in minced meat samples.

Considering that a specific limitation for a number of lactic acid bacteria does not exist, the summarizing distribution of lactic acid bacteria determination was individually presented in Figure 1. It can be observed that all tested samples had the presence of LAB. The LAB is part of the normal meat microbiota, and contamination with this bacterial group is expected at some level.

**Figure 2:**
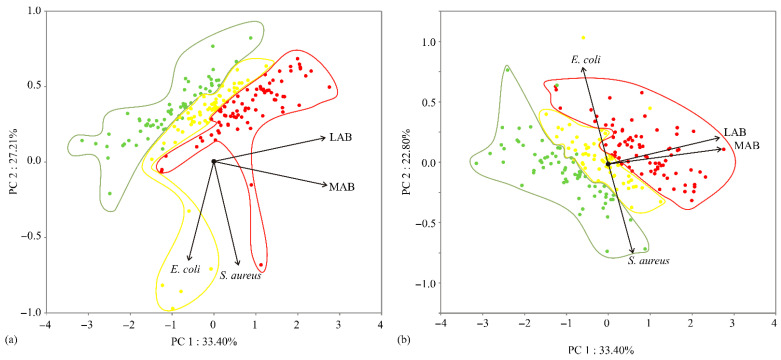
PCA ordination of variables based on component correlations: (**a**) projection in PC1-PC2 plane, (**b**) projection in PC1-PC3 plane for mesophilic and aerobic bacteria (MAB), *E. coli*, lactic acid bacteria (LAB), and *S. aureus*.

Additionally, The PCA of the presented data (Figure 2) explained that the first three components accounted for 80.10% of the total variance (33.40, 27.20, and 22.80%, respectively) in the four quantitative variables’ factor space: mesophilic and aerobic bacteria (MAB), *E. coli*, lactic acid bacteria (LAB), and *S. aureus*. From this statistical analysis, *Salmonella* spp. and *L. monocytogenes* are excluded, because they were not influential in any of the tested samples. For a better understanding of the obtained results, each gained result is classified using different colours based on levels represented in the Rulebook and the *Guide to Microbiological Criteria for Food*. The green colour represents a satisfactory level of the obtained results, while yellow and red signify an acceptable and unsatisfactory level of tested samples, respectively.

Based on the previously presented data about the microbiological quality profile of the meat samples, Table 3 provides information about the obtained artificial neural network (ANN). For the first time, a microbiological profile of beef samples was evaluated through this advanced mathematical analysis. According to ANN performance, it was noticed that the optimal number of neurons in the hidden layer for aerobic and mesophilic bacteria, *E. coli*, LAB, and *S. aureus* prediction was equal to 10 (network MLP 9-10-4) to obtain high values of *r*^2^ (overall *r*^2^ was 0.867 during training period) and low values of SOS (Table 3). The obtained coefficients of determination for training, testing, and validation sequences of ANN modelling were 0.867, 0.832, and 0.854, respectively. This indicates the accuracy of the proposed ANN model. Furthermore, the Broyden–Fletcher–Goldfarb–Shanno (BFGS) 6447 was used as a training algorithm, and the error function was SOS (sum of squares). The optimal hidden and output layer activation functions were logistic and identity, respectively. It can be concluded that the gained ANN model (MLP 9-10-4) predicted experimental variables reasonably well for a broad range of variables (Table 3).

**Figure 3:**
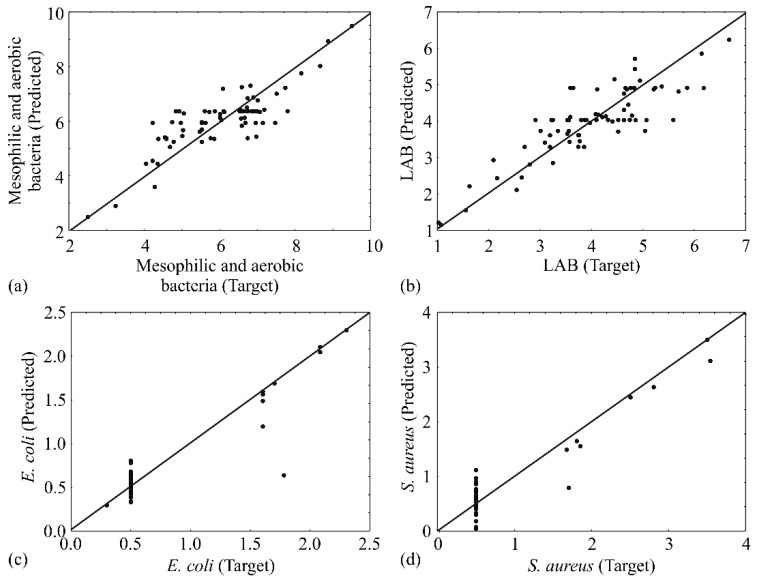
Experimentally gained and the ANN predicted values of the microbiological profile of minced meat: (**a**) Mesophilic and aerobic bacteria, (**b**) LAB. (**c**) *E. coli*, (**d**) *S. aureus*.

The accuracy of the ANN model could be visually assessed by the dispersion of points from the diagonal line in the graphics presented in Figure 3. The predicted values were very close to the desired values in most cases in terms of the value of the coefficient of determination.

Table 4 presents the elements of matrix *W*1 and vector *B*1 (presented in the bias row), as well as the elements of matrix *W*2 and vector *B*2 (bias) for the hidden layer, used for calculation in Equation (1).

As a statistical test that determines how well sample data fit a distribution from a population with a normal distribution, numerical verification of the obtained data was conducted. The goodness of fit, between experimental measurements and calculated results, was presented in Table 5. For this quality analysis, the following parameters were presented: reduced chi-square (χ^2^), root mean square error, (RMSE), mean bias error (MBE), coefficient of determination (*r*^2^), skewness (Skew), kurtosis (Kurt), mean of the residuals (Mean), standard deviation of the residuals (SD), and variance of the residuals (Var.). Except for the quality of the model fit, the residual analysis of the developed predictive model was presented in the same table. The presented four-parameter sigmoidal mathematical model appears to be simple, robust, and accurate. Mathematical models had an insignificant lack of fit tests, which means that all the models represented the data satisfactorily. A high coefficient of determination (*r^2^)* is indicative that the variation was accounted for and that the data fitted adequately to the proposed model. The adequacy of the model can be summarized by comparing the microbiological parameters as follows (in descending order): *S. aureus*, *E. coli*, lactic acid bacteria, and mesophilic and aerobic bacteria (Table 5). The obtained results indicate the possibility of using the ANN model in the presented experimental setup.

**Figure 4:**
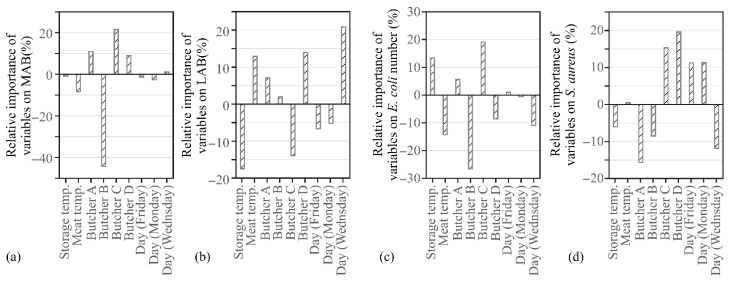
The relative importance of the input variables determined by the Yoon interpretation method: (**a**) Mesophilic and aerobic bacteria, (**b**) LAB. (**c**) *E. coli*, (**d**) *S. aureus*.

For understanding the influence of input variables on the microbiological profile of beef minced meat, global sensitivity analysis (Yoon’s interpretation method) was used. The obtained results are presented in Figure 4.

## 4. Discussion

According to the following Rulebook [29] and the Guide [30], microbiological criteria for food can be divided into two significant groups. Briefly, the food safety criterion is applied to assess the safety of a product or production batch, and this criterion applies to products during product shelf-time. If food safety criteria are not satisfied, the tested product cannot be placed on the market or have to be withdrawn/recalled from the market [30]. The second criteria include the criterion of hygiene in the production process, which indicates correct production processing. If this criterion is not fulfilled, the applied procedures should be reviewed, and corrective measures should be applied in order to improve the hygiene of the production process. Both microbiological criteria for food are determined based on the general principles of international standards and guidelines on food safety (Codex Alimentarius), conclusions drawn up by the Scientific Committee on Veterinary measures relating to Public Health [30] as well as a timeline of the actual European regulations.

In order to test both food safety criteria as well as criteria of hygiene, this study involves the determination of the microbiological profile of freshly processed minced meat based on *Salmonella* spp. and *Listeria monocytogenes* (food safety criteria) and mesophilic and aerobic bacteria (MAB), *Escherichia coli*, and *Staphylococcus aureus* (criteria of hygiene). Additionally, lactic acid bacteria (LAB) as one of the specific spoilage groups of microorganisms for meat are tested [25]. In this way, requirements related to the product during its shelf life, as well as the possibility of improving production hygiene and the choice and/or origin of raw materials are included in the following analyses. According to the obtained results of microbiological analysis (Table 2), in the case of analysis of mesophilic and aerobic bacteria (MAB), only 32.85% of samples were at a satisfactory level, while 26.85% were at an acceptable level. In the rest of the samples, approx. 40% of samples, were unsatisfactory because the number of detected aerobic and mesophilic bacteria was above 6 log CFU/g. This group of bacteria presents a practical problem in meat production and for butchers. Many scientific papers reported a high level of aerobic and mesophilic bacteria in minced meat, which is unsatisfactory and presented a big challenge in view of the reduction in microbiological contamination in order to prolong shelf-life [10,11,23,24]. Briefly, the obtained results in this investigation are in agreement with the results by Siriken [10], who reported 56% of samples with an unsatisfactory level of tested bacteria. A similar result is observed by Zerabruk et al. [11], where 67% of minced meat samples contained an unsatisfactory level of these bacteria. The trend of MAB multiplication was detected as a function of time [23], so only freshly- and cold-storage processed meat can be acceptable for consumption. 

Out of the total number of meat samples, more than 95% of samples were satisfactory in view of *E. coli* analysis with an acceptable level according to the Guideline. The minced meat contaminated with *E. coli* can be a source of several illnesses, including haemolytic uremic syndrome, haemolysis, thrombocytopenia, renal failure, etc. [24]. When *E. coli* is detected in meat products, meat production needs to revise current hygiene practices and provide a safer product. *L. monocytogenes* were not noticed in any of the 216 samples, while *Salmonella* spp. was present in only 6.9% of samples. The non-appearance of *Salmonella* spp. is especially important considering the widespread presence of *Salmonella* in minced meat, salmonellosis outbreak, and the frequency of its consumption via many traditional products [2]. Furthermore, meat products are frequently observed as one of the main sources of listeriosis caused by *L. monocytogenes*, especially by the three serotypes 1/2a, 1/2b, and 4b [43]. On the other hand, the absence of *L. monocytogenes* in the samples after the production process does not provide security for recontamination during final packaging or later handling [44]. In the case of *S. aureus* contamination, the total percent of samples at on satisfactory level was 94%, which is a greater value than previously reported (62%) by Shawish and Al-Humam [20] as well as by Saad et al. (between 28 and 60%) [45].

Additionally, Figure 1 indicates that the most frequent ranges of LAB concentration were 2–4 log CFU/g and 4–6 log CFU/g, while ranges of 0–2 and 6–7 log CFU/g were spotted in the case of several samples annually. The scientific literature emphasizes that the number of LAB above 7 log CFU/g leads to the development of spoilage and results in meat unacceptability [46], wherefore the obtained results in this study are propitious. On the other hand, the presence of LAB can have a positive effect on the meat. Namely, metabolic activities and produced metabolites can have an inhibitory effect on food spoilage and pathogenic bacteria such as *E. coli* and *S. aureus* [46].

The points in the PCA graphics (Figure 2), which are in close vicinity to each other, display the possible similarity of patterns that are assigned to these points. The orientation of the vector which describes the variable in factor space indicates an increasing trend of the variable, while the length of the vector is proportional to the square of the correlation coefficient between the fitting value for the variable and the variable itself. The angles between corresponding vectors indicate the value of the correlation coefficient between these variables (small angles corresponding to high correlations). Considering the map of the PCA performed on the data, aerobic and mesophilic bacteria and LAB (which contributed 47.2 and 48.2% of the total variance, respectively, based on correlations) positively influenced the first-factor coordinate (PC1), while *S. aureus* (49.8%) and *E. coli* (49.8%) exhibited negative scores according to the second principal component (PC2). *E. coli* (51.2%) showed a positive influence on the third principal component, while *S. aureus* (43.8%) showed a negative influence toward the third principal coordinate (PC3) (Figure 2).

Many empirical models are used for understanding microbiological profiles of meat and meat products, but a step forward in advanced mathematical and statistical modelling is still not widely applied. Only a few studies included using predictive tools for an explanation of microbiological experimental data, such as partial least squares regression analysis [47], growth kinetics modelling [48,49], nonlinear least square regression analysis with the Levenberg–Marquardt method of estimation [50], Lorentzian distribution function and Nonlinear regression [51], etc. On the other hand, when attempting to build a mathematical model in the area of predictive microbiology, the artificial neural network (ANN) model is perceived as one of the most relevant and universal anticipating tools [52,53]. In this investigation, these facts are used and verified for the first time for analysing the obtained results of microbiological profiling of meat samples. The elaborated optimal ANN model exhibited adequate generalization ability to the acquired experimental data prediction and can be utilized to foresee the precise output for a wide span of the input parameters. The anticipated values were in close vicinity to the expected values in most cases, in relation to the *r*^2^ value, for ANN models. The ANN model anticipated experimental aerobic and mesophilic bacteria, *E. coli*, LAB, and *S. aureus* rather well for a broad range of the parameters, as seen in Figure 3, where the experimentally obtained and ANN model anticipated values are shown. Based on the quality parameters displayed in Table 4, the ANN model had a negligible lack of fit tests, meaning that the ANN model presented the data correctly. Additionally, the goodness of fit among experimental data and ANN model results was displayed in Table 5. A high *r*^2^ is illustrative that the data fitted satisfactorily to the developed model.

According to Figure 4, which presents the Yoon sensitivity model, Butcher C was the most important factor for predicting the MAB of beef minced meat, with a relative importance of 21.48%, while the influence of Butcher B exerted the most negative impact, with −44.32% negative importance. Weekdays (Wednesday) were the most important factor for predicting the LAB of beef minced meat, with a relative importance of 20.90%. while the influence of storage temperature showed the most negative impact, with −17.44% negative importance. Butcher C was the most important factor for predicting the number of *E. coli* in beef minced meat, with a relative importance of 19.08%, while the influence of Butcher B showed the most negative impact, with −26.62% negative importance. Butcher D was the most important factor for predicting the amount of *S. aureus* in beef minced meat, with a relative importance of 19.60%, while the influence of Butcher A showed the most negative impact, with −15.67% negative importance. Due to the obtained results of the relatively high influence of butcher shift on the microbiological quality of meat, it can be suggested that certain workers maintain much more hygiene in the working space than others, doing the work in accordance with good hygienic practice and in the shortest possible time. Accordingly, it would be recommended that a specific aspect of corrective action be taken in relation to these results. Although storage and meat temperature do not have a high relative influence on the number of MAB, these two variables have a great influence on other tested microorganisms. Consequently, it can be seen in Figure 4 that meat and storage temperature have different relative influences depending on LAB, *E. coli*, and *S. aureus*. 

In the meat industry, all production, storage, and transport processes need to be under cold conditions, in a temperature range between 2 and 7 °C [54]. Frequently, incorrect temperatures have been reported as an essential problem for meat quality [55]. Variability in storage and meat temperatures can be very problematic because it can decrease the shelf-life of meat, which almost no producer will not estimate when determining an expiration date. Taking into account these parameters as one of the essentials to determine the shelf-life of meat, predictive modelling can be a very important tool to determine a definitive remaining self-life, upgrade storage conditions, and minimize economic loss.

## 5. Conclusions

A parallel mathematical investigation of feasible analysis and the construction of a mathematical model displayed suitable accuracy for the anticipation of the quality of minced beef meat, which is one of the most consumable types of meat, but also a food product with short shelf life due to propensities for microbial growth. According to the obtained results, all tested samples had a relatively high number of aerobic and mesophilic bacteria, while *E. coli, Salmonella* spp., and *S. aureus* appeared randomly during the examination period. The butcher shift and temperature were the most important factors for predicting the microbiological profile of beef minced meat, with a high relative importance of tested microbiological parameters. Furthermore, the developed artificial neural network provided a good prediction of the microbiological profile of beef minced meat with an overall *r*^2^ of 0.867 during the training cycle. It can be concluded that maintenance of good hygiene practices in a meat production and distribution process can prevent increasing the number and occurrence of some microorganisms, but potential critical points are already in the primary production process. In terms of practicality, ANN models offer a simpler alternative to traditional models for the prediction of microorganisms but require a greater amount of parameters and data. Furthermore, using statistical analysis of the gained microbiological results in the everyday routine of meat processing can facilitate the establishment of microbiological quality control system and minimize economic losses. In future work, it is recommended to carry out the prediction model of other indicators of the physicochemical and microbiological quality of meat for other meat industries based on the proposed methodology. In addition, it is recommended to extend the prediction model to the supply chain, taking into account other types of storage and exploitation categories. For example, all parameters in maintaining hygiene during production and the whole transport chain can be based on a better understanding.

## Figures and Tables

**Table 1:** ISO methods for microbiological analysis.

Microbiological Analysis of Minced Meat		Allowable Limit Values (m- and M-Values) According to the Guide to Microbiological Criteria for Food (log CFU/g)
Microorganism/Group of Microorganisms	Method Ref.	
Mesophilic and aerobic bacteria (MAB)	[31]	m = 5; M = 6
*Escherichia coli*	[32]	m = 2; M = 3
*Salmonella* spp.	[33]	nd *
*Listeria monocytogenes*	[34]	nd
*Staphylococcus aureus*	[35]	m = 2; M = 3
Lactic acid bacteria (LAB)	[36]	**

* nd—not detected in 25 g of samples; ** the *Guide to Microbiological Criteria for Food* did not indicate a value for this parameter.

**Table 2:** The descriptive analysis of minced meat samples.

Microbiological Analysis	Allowable Limit Values (m- and M-Values) *	The Average Percentage (%) of Samples Classified as:		
		Satisfactory	Acceptable	Unsatisfactory
Mesophilic and aerobic bacteria (MAB)	m = 5; M = 6	32.85	26.85	40.3
*E. coli*	m = 2; M = 3	95.4	4.6	0
*Salmonella* spp.	nd **	93.1	/	6.9
*L. monocytogenes*	nd	100	/	0
*S. aureus*	m = 2; M = 3	94	3.7	2.3

* m- and M- values are indicated in log CFU/g; ** nd—not detected in 25 g of samples.

**Table 3:** ANN summary or observed results.

The Obtained Network Name	MLP 9-10-4	
Performance	Training	0.867
	Testing	0.832
	Validation	0.854
Error	Training	0.484
	Testing	0.490
	Validation	0.897
Training algorithm	BFGS 6447	
Error function	SOS	
Hidden activation	Logistic	
Output activation	Identity	

**Table 4:** Coefficients assigned to the hidden and the output layers (weights and biases).

Elements of Matrix *W*_1_ and Vector *B*_1_ (Presented in the Bias Row)											
Independent Variables	1	2	3	4	5	6	7	8	9	10	
Store temperature	3.70	0.37	−147.96	−17.66	−82.56	−15.34	231.07	29.54	28.94	−14.48	
Meat temperature	99.23	142.56	58.25	20.10	65.60	27.46	−94.96	105.16	151.15	25.96	
Butcher A	−65.60	−105.46	−37.80	−7.51	−20.39	−14.53	35.69	−78.06	61.40	−121.10	
Butcher B	107.49	303.75	29.99	2.40	20.45	2.62	−80.11	115.68	41.76	52.01	
Butcher C	41.19	−63.16	59.55	3.81	19.07	4.05	34.42	−64.03	−101.17	53.46	
Butcher D	−65.26	−103.04	1.93	2.01	14.19	7.24	−114.51	98.64	−34.24	56.38	
Weekday (Friday)	6.55	6.67	52.01	7.05	73.63	7.72	44.36	78.00	93.39	73.42	
Weekday (Monday)	4.87	10.37	25.22	−1.54	−2.84	0.28	−37.29	58.36	−73.23	66.34	
Weekday (Wednesday)	6.45	15.05	−23.48	−4.91	−37.44	−8.57	−131.65	−64.04	−52.44	−98.96	
Bias	17.80	32.05	53.77	0.68	33.38	−0.58	−124.66	72.23	−32.28	40.75	
Elements of matrix *W*_2_ and vector *B*_2_ (presented in the bias column)											
Output variables	1	2	3	4	5	6	7	8	9	10	Bias
MAB *	65.57	−65.39	−5.27	−0.52	5.61	5.91	−3.40	−0.25	0.08	−5.50	0.30
LAB **	3.41	−3.35	−38.85	0.96	38.44	15.05	−27.28	0.49	−0.15	−16.01	0.56
*E. coli*	55.53	−55.56	71.22	0.43	−71.60	2.03	50.36	0.16	−0.10	−2.21	0.09
*S. aureus*	20.40	−20.35	−13.56	−0.59	13.24	−33.94	−9.39	0.40	0.04	34.45	−0.06

* MAB—mesophilic and aerobic bacteria; ** LAB—lactic acid bacteria.

**Table 5:** The ‘goodness of fit’ tests for the microbiological profile prediction of beef minced meat.

Parameters	*χ* ^2^	RMSE	MBE	MPE	*r* ^2^	Skew	Kurt	SD	Var.
MAB *	1.612	1.176	0.000	26.823	0.592	−0.125	−0.274	0.738	0.545
LAB **	1.047	0.947	0.000	31.823	0.731	0.144	0.085	0.595	0.354
*E. coli*	0.087	0.2737	0.000	36.413	0.819	4.195	27.617	0.171	0.029
*S. aureus*	0.153	0.362	0.000	67.756	0.880	0.933	3.896	0.228	0.052

* MAB—mesophilic and aerobic bacteria; ** LAB—lactic acid bacteria; Abbreviations: *χ^2^,* reduced chi2 square; RMSE, root mean square error; MBE, mean bias error; MPE, mean percentage error; *r^2^*, coefficient of determination; Skew, skewness; Kurt, kurtosis; SD, the standard deviation of the residuals; Var, the variance of the residuals.

## Data Availability

Not applicable.
